# Supplementary material for: DTDHM: detection of tandem duplications based on hybrid methods using next-generation sequencing data
Source: PeerJ. 2024 Jul 26;12:e17748. doi: 10.7717/peerj.17748 (PMC11285389; doi:10.7717/peerj.17748)
Supplement: Supplemental Information 1 [file peerj-12-17748-s001.docx]

**Supplemental File**

1. **Analysis of Time and Memory Consumption**

We evaluate the runtime and memory consumption of DTDHM, SVIM, TARDIS, and TIDDIT. The data used is based on real data (NA19238) from chromosome 21 of the reference GRCh38. The sequence coverage depth is 6X. Figure 1.1 illustrates the runtime of the four methods, measured in seconds (s). Under the same constraints, SVIM demonstrates higher efficiency and superior performance. It can accomplish tasks more quickly, with a runtime of only 11s. The runtime of DTDHM ranks second at 42s. TARDIS and TIDDIT exhibit the slowest execution speeds.


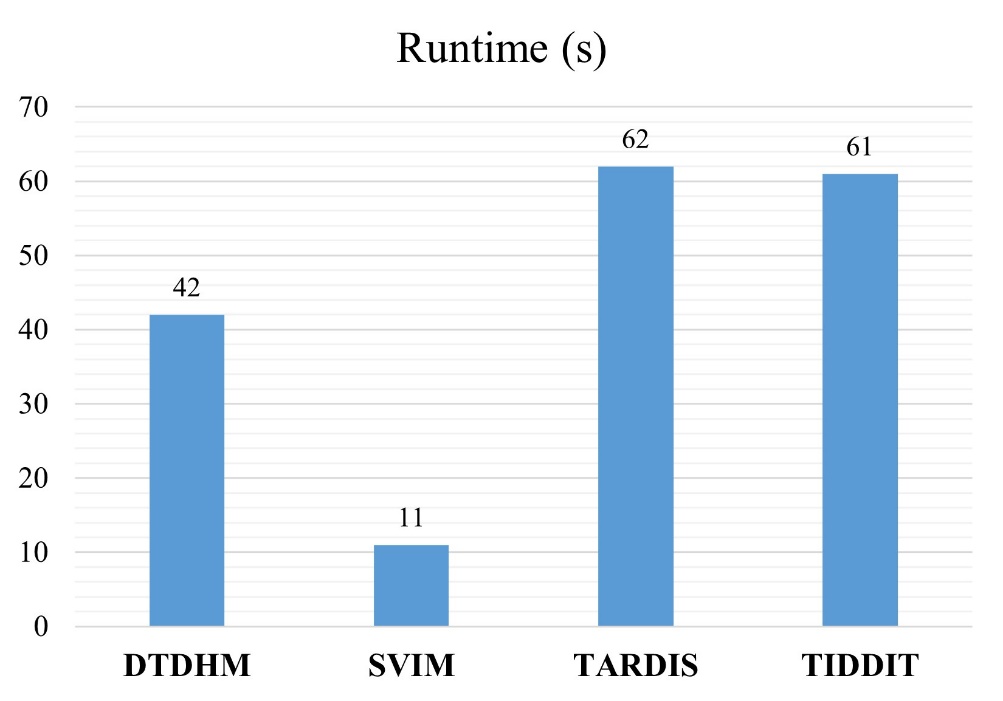


**Figure 1.1.** The runtime of the four methods, measured in seconds (s).

Table 1.1 displays the memory consumption of the four methods. Where, VIRT represents the virtual memory usage (in Gigabytes); RES represents resident memory usage (in Gigabytes); SHR represents shared memory usage (in Megabytes); MEM represents percentage of memory usage. From the data in the Table 1.1, it can be observed that DTDHM has relatively low memory consumption, with an actual physical memory (RES) of only 1.2 GB, and a low memory usage rate. This indicates that DTDHM is designed to utilize memory more efficiently. TARDIS has the highest memory consumption, with its RES being 6.6 GB, occupying most of the system's physical memory. Its virtual memory (VIRT) is also substantial, reaching 6.6 GB.

**Table 1.1.** The memory consumption of the four methods.

| Method | VIRT (GB) | RES (GB) | SHR (MB) | MEM (%) |
| --- | --- | --- | --- | --- |
| DTDHM | 4.4 | 1.2 | 97.1 | 8.1 |
| SVIM | 1.7 | 0.1 | 36.4 | 0.5 |
| TARDIS | 6.6 | 6.6 | 2.8 | 42.7 |
| TIDDIT | 6.7 | 3 | 22.2 | 19.7 |

1. **Parameter Values**

In the simulated data, we select the optimal value for each parameter through experimental validation and empirical judgment. Three configurations are randomly selected for each parameter, and for each configuration, five sets of data are generated. Subsequently, the average F1 score for each configuration is calculated for comparison.

(1) The length of genome bin ($L_{b}$)

The length of genome bin is generally measured in 500bp, 1000bp and 2000bp. Figure 2.1 shows the impact of different values of $L_{b}$ on the F1-score. As can be seen from the figure, different values of $L_{b}$ greatly affect the detection result. For example, in the 6X-0.4 sample, F1-scores with $L_{b}$ of 1000bp are 6.6% and 8.35% larger than those with $L_{b}$ of 500bp and 2000bp, respectively. Experimental results show that DTDHM method has a good detection effect when $L_{b}$=1000bp.


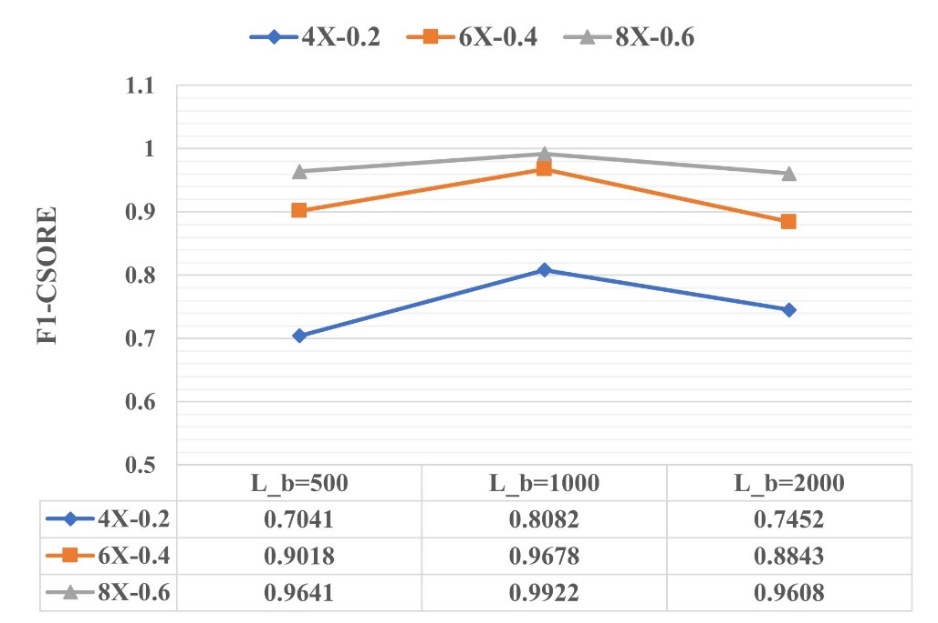


**Figure 2.1.** The impact of different values of $L_{b}$ on the F1-score.

(2) The parameter *k* in the KNN algorithm

In the tandem duplication detection, finding an appropriate value for *k* can be challenging. Therefore, we specified a range of values [0.05$N_{r}$, 0.3$N_{r}$], and compared their F1-scores to determine the final value of *k*, as shown in Figure 2.2. When *k* is greater than or equal to 0.2$N_{r}$, the F1-score reaches its peak and stabilizes. Additionally, considering that smaller values of *k* result in faster KNN search speeds, we default to using 0.2$N_{r}$ as the value of *k*.


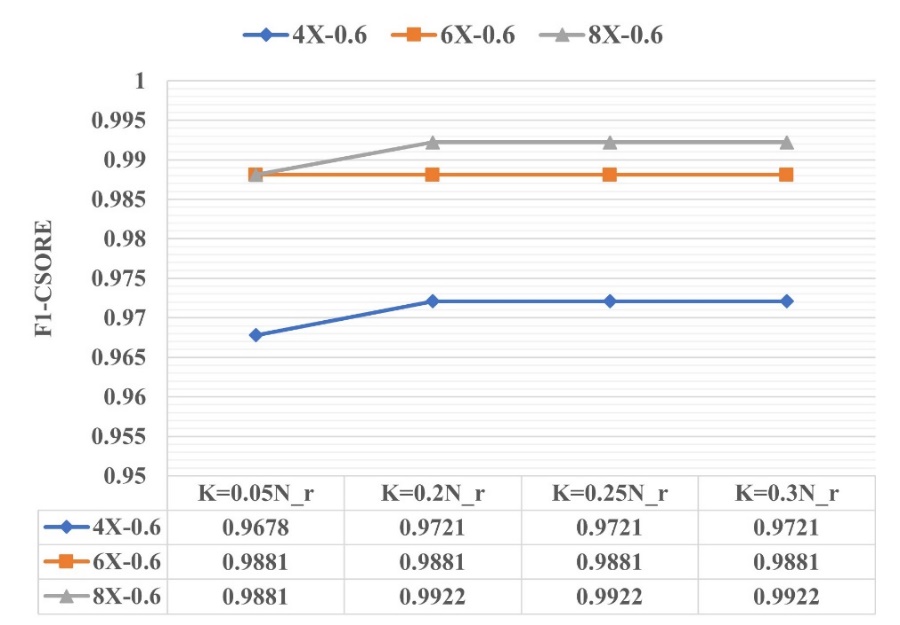


**Figure 2.2.** The impact of different values of *k* on the F1-score.

(3) The parameter *θ* in the boxplot.

The parameter *θ* is an important parameter in DTDHM, used to regulate the threshold of outlier scores. By utilizing this threshold, we can filter out potential tandem duplications, and its value will affect the number of tandem duplications correctly predicted by the DTDHM method. In the simulated data, the F1 score of the configuration *θ*=0 is slightly higher than that of *θ*=0.6. Furthermore, considering the complexity of data in real samples and the need to ensure the stability of the DTDHM method under various circumstances, we set the default value of parameter *θ* to 0.6. We allow users to adjust the value of parameter *θ* themselves, and we recommend a range of [0, 1].


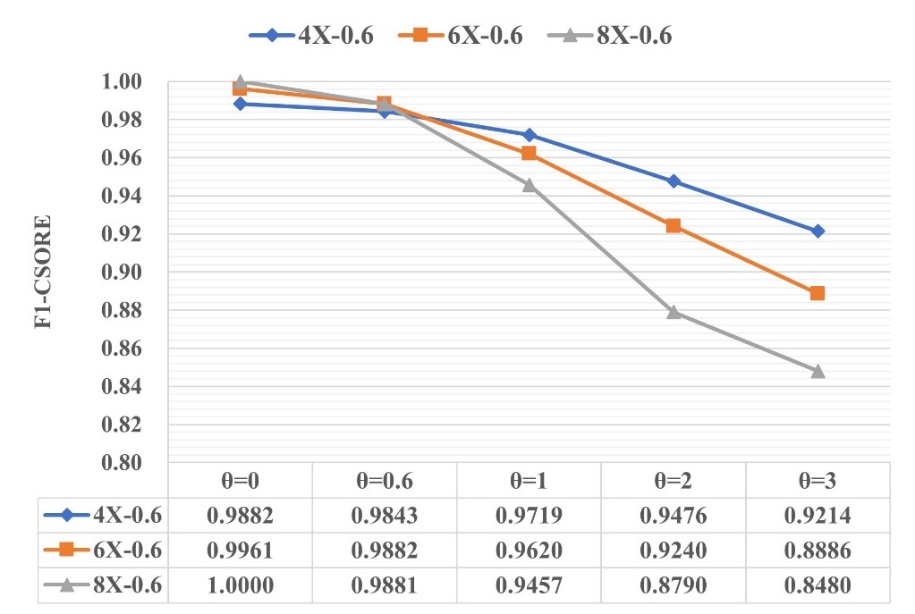


**Figure 2.3.** The impact of different values of *θ* on the F1-score.

1. **Detection of the method in the 10X_0.6 configuration**

We generated 50 sets of simulated data with the configuration 10X_0.6, and calculated the F1-scores and boundary biases of each method, as shown in Figure 3.1. From Figure 3.1(A), it can be observed that the F1-score of the DTDHM method is 98.60%, ranking first, followed by TIDDIT with 81.24%, TARDIS with 72.95%, and SVIM with 61.60%. In Figure 3.1(B), the average boundary biases of DTDHM, SVIM, TARDIS, and TIDDIT on the 50 sets of data are 26.84bp, 42.31bp, 110.12bp, and 53.24bp, respectively. The boundary bias of the DTDHM method remains the best in the 10X_0.6 configuration, ranking first. The boundary accuracy of TARDIS detection results is the worst. The experimental results demonstrate that DTDHM still achieves good performance under the 10X_0.6 configuration.


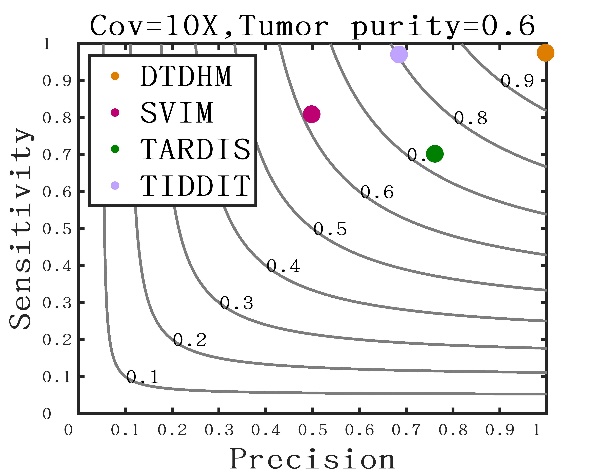

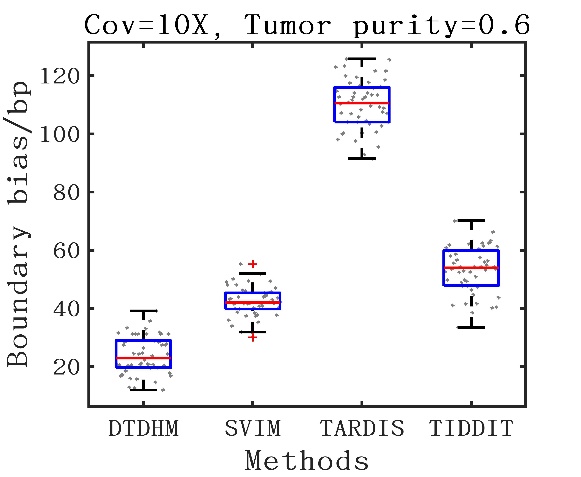


(A) (B)

**Figure 3.1.** Detection of the method in the 10X_0.6 configuration.
